# Clear Cell Carcinoma Arising in Vulvar Endometriosis

**DOI:** 10.1155/2018/4263104

**Published:** 2018-07-26

**Authors:** Pranom Buppasiri, Pilaiwan Kleebkaow, Chantip Tharanon, Apiwat Aue-aungkul, Chumnan Kietpeerakool

**Affiliations:** Department of Obstetrics and Gynaecology, Faculty of Medicine, Khon Kaen University, Thailand

## Abstract

We report a case of vulvar clear cell adenocarcinoma in a woman presenting with a lump and pain in the right side of the labia majora. Three years prior to this visit, she underwent a total abdominal hysterectomy with bilateral salpingooophorectomy and excision of a labial mass. Pathological examination revealed adenomyosis and multiple leiomyomas in the uterus, endometriotic cysts in both ovaries, and foci of atypical endometriosis in the labial mass. The results of an incision biopsy performed before referral indicted only apocrine hidrocystoma. Physical examination revealed a hard mass at the right labia majora extending to the right groin. The mass seemed to be in continuity with the pubic symphysis that would require pubic bone excision and reconstruction with flap surgery to achieve complete resection. However, the patient refused such extensive surgery. Based on previous diagnosis of vulvar endometriosis, she had been treated with GnRH agonists and depot medroxyprogesterone acetate. However, the mass developed into an ulcer and increased in size. A second biopsy of the mass was undertaken, and the pathological diagnosis was clear cell carcinoma with coexisting atypical endometriosis. Computed tomography of the abdominopelvic region showed an ulcerative mass at the right labia majora and nodal metastasis at the external iliac and inguinal regions. Systemic chemotherapy was administered. The growth of the tumors stabilized during the first two cycles of chemotherapy but rapidly progressed thereafter. At 17 months after her initial presentation, the patient passed away due to the progression of the disease.

## 1. Introduction

Endometriosis is characterized by the presence of endometrial glands and stroma similar to that in the endometrial lining, but in a location outside of the uterine cavity [1]. Endometriosis can be further classified as either endopelvic or extrapelvic depending on the location of the endometrial tissue implantation [1]. Endopelvic endometriosis commonly involves the ovaries, fallopian tubes, uterosacral ligament, pouch of Douglas, and rectovaginal septum. Endometriosis deposits can also occur in the gastrointestinal tract, thoracic cage and lungs, diaphragm, nervous system, and mucocutaneous tissue [1]. A previous study found the prevalence of extrapelvic endometriosis to be 20% in women with endometriosis [2]. In that study, the gastrointestinal tract was the most common site of extrapelvic endometriosis (52%), followed by the urinary tract (35%). In addition, approximately 78% of women with extrapelvic endometriosis also had pelvic endometriosis [2].

Women with endometriosis have an increased risk of certain histologic types of cancer, which mostly involve the ovary [3]. In the recent model of epithelial ovarian carcinogenesis, endometriosis has been proposed as a precancerous lesion of endometrioid, clear cell, and seromucinous carcinomas of the ovary [4]. The malignant transformation of extrapelvic endometriosis is extremely rare [3]. Therefore, an accumulation of case reports regarding this uncommon clinical entity is necessary in order to verify its clinical course and predisposing factors. Herein, we present a rare case of a patient with clear cell carcinoma of the vulva related to neoplastic transformation of endometriosis who presented with a painful mass at the right side of the groin and labia major.

## 2. Case Presentation

A 46-year-old nulliparous woman presented after having experienced pain in the right side of her groin and labia majora for four months, as well as a lump that was increasing in size. She had previously undergone three laparotomies for ovarian cystectomy at 20, 10, and 8 years prior to this visit due to her severe progressive pelvic pain. The pathological examinations of surgical specimens obtained from all three operations indicated ovarian endometriosis.

**Figure 1:**
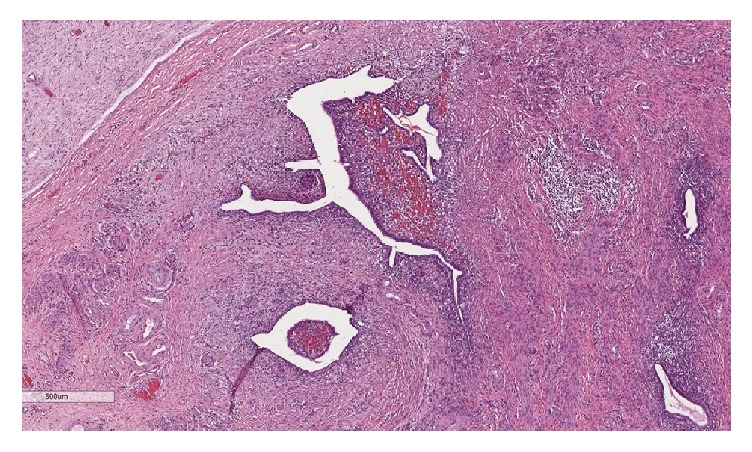
Microscopic examination of the labial mass reveals multiple foci of endometriosis (4x).

**Figure 2:**
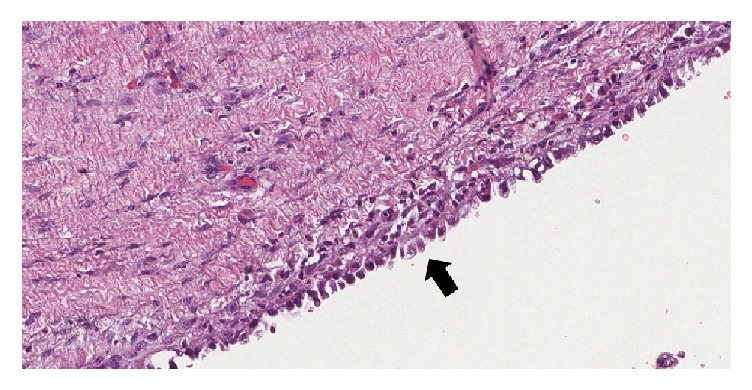
Microscopic examination shows a focus of endometriosis in the labial mass that lined by endometrial cells with cytologic atypia (10x).

Three years prior to this visit, she experienced progressive pelvic pain and she also noticed a growing mass at the right labia majora. She underwent total abdominal hysterectomy (TAH) with bilateral salpingooophorectomy (BSO) and excision of a 3.7 × 2.5 cm labial mass. Pathological examination revealed adenomyosis and multiple leiomyomas in the uterus as well as endometriotic cysts in both ovaries. The labial mass contained focal atypical endometriosis on a background of benign endometriosis (Figures 1 and 2). The patient had an uneventful recovery. However, she did not return to follow-up after this operation. She had no history of hormonal replacement therapy (HRT).

One month before this visit, she had undergone an incision biopsy of her labial mass at the provincial hospital, and the pathological report indicted only apocrine hidrocystoma. Nevertheless, the mass had increased in the size with accompanying progressive pain.

**Figure 3:**
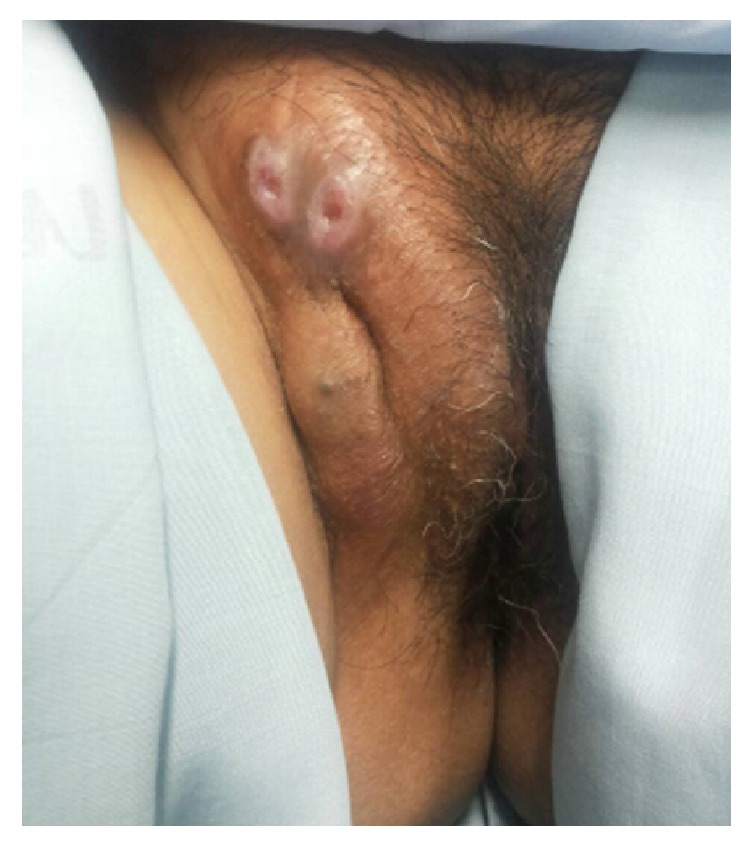
A hard consistency mass at the right labia majora extending to the right groin with ulceration.

**Figure 4:**
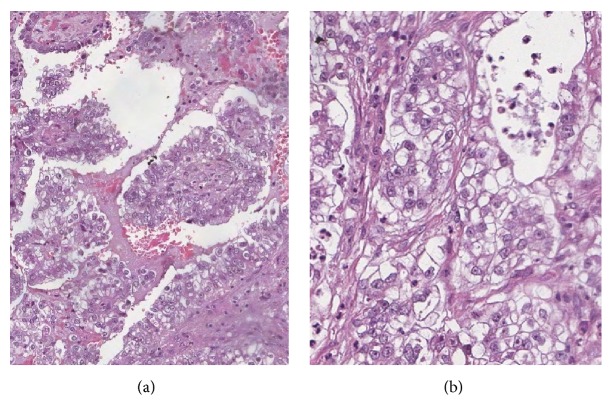
Microscopic examination displays the papillary growth structures lined by round tumor cells with clear cytoplasm and pleomorphic nucleoli (4x(a) and 10x (b)).

**Figure 5:**
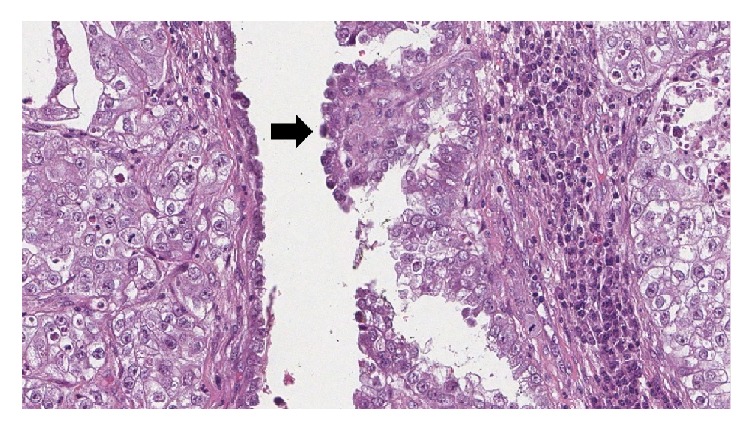
Microscopic examination shows part of a tiny cyst lined by cuboidal cells with atypical nuclei that had merged with the tumor indicating residual foci of atypical endometriosis (10x).

Upon presentation at our hospital, there was a 7x4-cm hard mass at the right labia majora extending to the right groin area. The mass seemed to be in continuity with the pubic symphysis that would require pubic bone excision and reconstruction with flap surgery to achieve complete resection. However, the patient refused such extensive surgery. Based on the previous pathological diagnosis of endometriosis of the vulva in this patient, she was then initially treated with two doses of GnRH agonist followed by one 150 mg dose of depot medroxyprogesterone acetate. These medications were administered with the aim of shrinking the bulk of the mass. However, the mass developed into an ulcer and rapidly increased in size (Figure 3). A second biopsy of the mass was then performed. The pathologic examination showed a papillary growth structure lined by round tumor cells with clear cytoplasm and pleomorphic nucleoli (Figure 4).  Figure 5 shows residual atypical endometriosis that had merged with the tumor. The patient's tumor exhibited focal cytoplasmic staining for napsin A. A diagnosis of clear cell adenocarcinoma of the vulva with coexisting atypical endometriosis was made based on these pathological findings.

Computed tomography of the abdominopelvic region showed an ulcerative mass at the right labia majora and nodal metastasis at the bilateral external iliac and superficial inguinal regions that varied in size from 2.0 to 3.5 cm. Other CT findings were unremarkable.

As this case was considered inoperable, systemic chemotherapy consisting of paclitaxel and carboplatin was administered. The growth of the tumors stabilized during the first two cycles of chemotherapy but rapidly progressed thereafter. Palliative treatment under multidisciplinary care was subsequently initiated. Pelvic radiation was administered to slow down the progression of disease and to alleviate pelvic pain. At 17 months after her initial presentation, the patient passed away due to the progression of the disease and severe sepsis.

## 3. Discussion

We report a case of vulvar clear cell carcinoma in a 46-year-old nulliparous woman who presented with a four-month history of a lump and pain in the right side of her groin and labia majora. Clear cell carcinoma of the vulva is a rare phenomenon and the most likely mechanism in this case is malignant transformation of residual foci of atypical endometriosis within the vulva.

Although the pathogenesis of malignant changes in endometriosis remains unclear, hyperestrogenism has been proposed as one of the potential risk factors based on the fact that there are some reported cases in which endometriosis-associated adenocarcinoma developed in the large bowel, bowel mesentery, peritoneum, and vagina during estrogen replacement therapy after TAH with BSO [5–10]. The duration of unopposed estrogen therapy in these reports varied from 22 months to 17 years [5–10]. Interestingly, the pathology in these cases was endometrioid adenocarcinoma [5–10]. However, there is currently no evidence of an association between hyperestrogenism and the occurrence of endometriosis-associated clear cell adenocarcinoma. We believe that the malignant transformation of endometriosis may progress along one of two possible pathways. As endometriosis is an ectopic endometrium, hyperestrogenism may stimulate the transformation of endometriosis into atypical hyperplasia and endometrioid adenocarcinoma, similar to the process by which endometrioid adenocarcinoma has been found to develop in the endometrium [11]. The other possibility is that epithelial atypia arises in an endometriotic lesion and then evolves into clear cell carcinoma, similar to the development of clear cell carcinoma of the ovary [12, 13]. In our patient, pathological examination of the vulvar specimen obtained from her previous operation revealed foci of atypical endometriosis. There was also a tiny cyst merged with clear cell carcinoma, which was suggestive of atypical endometriosis in the vulvar incision biopsy specimen. In addition, our patient had no history of HRT after TAH with BSO.

Currently, the research is inconclusive with regard to the mechanisms that lead to the development of extrapelvic lesions. Extrapelvic endometriosis can occur via iatrogenic seeding of endometrial tissue at surgical sites during labor or an operation in which the endometrial cavity entered such as cesarean section or myomectomy. This accounts for the presence of endometriosis in previous episiotomy sites and abdominal wall scars from cesarean sections. Other explanations for the spread of endometrial tissue to the outside of the pelvis include (i) the transportation of viable endometrial tissue in circulating fluid within the abdominal cavity, (ii) lymphatic or hematogenous dissemination, and (iii) the direct extension from pelvic endometriosis to extrapelvic areas [1].

In our patient, the lesion involved the right side of groin and labia majora. The occurrence of endometriosis in the vulva in our patient, which subsequently transformed to clear cell carcinoma, may have extended from the pelvis via the round ligament. Though extremely rare, the occurrence of endometriosis in the extrapelvic portion of the round ligament has been reported, and it involves the right side in more than 90% of cases [14–17]. One possible explanation for this rare clinical presentation is the presence of atypical lymphatic flows from the peritoneal cavity and pelvis to the right groin or nonobliteration of the parietal peritoneum accompanying the round ligament in the canal of Nuck, thereby enabling the viable endometrial tissue in the peritoneal fluid to deposit along the round ligament [18].

Due to the rarity of endometriosis-associated cancers, the optimal treatment modalities in these cases are not yet known. Complete surgical removal remains the most common treatment in cases of malignant transformation of endometriosis. Neoadjuvant chemotherapy may allow for a complete resection to be performed in some cases [19]. In cases with later presentation, adjuvant platinum-based chemotherapy following tumor debulking is commonly prescribed to minimize the risk of recurrence. However, the prognosis of patients with advanced endometriosis-associated cancer is generally poor because of the intrinsic chemoresistance-related gene of this cancer [20]. In our patient, surgical excision was not technically possible due to the disease being in its advanced stages. A systemic chemotherapy consisting of paclitaxel and carboplatin administered as neoadjuvant treatment was not effective. The time from presentation until death was 17 months.

## 4. Conclusion

Clear cell adenocarcinoma of the vulva is a rare phenomenon and the most likely mechanism in this case was malignant transformation of residual foci of atypical endometriosis within the vulva. Our report confirms the relatively poor prognosis of this type of tumor, particularly in cases of later presentation.
